# A humanized mouse model of APOB deficiency recapitulates hypobetalipoproteinemia

**DOI:** 10.1016/j.jlr.2026.101142

**Published:** 2026-09-02

**Authors:** Amita Tiyaboonchai, Anne Vonada, Jeffrey Posey, Kevin L. Keys, Carl Pelz, Helen Chen, Xiping Cheng, Markus Grompe

**Affiliations:** 1 Oregon Stem Cell Center, Oregon Health & Science University, Portland, OR, USA; 2 Papé Family Pediatric Research Institute, Department of Pediatrics, Oregon Health & Science University, Portland, OR, USA; 3 Bioinformatics and Computational Biology, Ambys Medicines, South San Francisco, CA, USA; 4 Regeneron Pharmaceuticals, Inc., Tarrytown, NY, USA

**Keywords:** abetalipoproteinemia, liver, cholesterol/metabolism, apolipoproteins, lipoproteins, dyslipidemia, apolipoprotein b, liver humanization, hypercholesterolemia, MASLD, gene editing

## Abstract

Lipoprotein metabolism is significantly different between mice and humans thus making it difficult to model disorders of human lipid metabolism in transgenic mice. Systemic lipoprotein metabolism is predominantly governed by hepatocytes, and mice with humanized livers display human-like lipid profiles. Here we report a highly efficient method to knock out genes in human hepatocytes while retaining their ability to repopulate immune-deficient rodents. As proof-of-principle, Fah-deficient, immune-compromised mice were repopulated with Apolipoprotein B (APOB) knockout human hepatocytes. Mice humanized with knockout cells recapitulated typical features of human hypobetalipoproteinemia. We conclude that that human genetic disorders of lipid metabolism can be accurately modeled in a chimeric liver mouse by utilizing human knockout hepatocytes.

Apolipoprotein B (APOB) is a secretory glycoprotein that plays a key role in lipoprotein transport and metabolism. In humans it exists as two distinct isoforms, APOB-100 and APOB-48. Both isoforms are encoded by a single gene, and APOB-48, the shorter isoform, is produced by RNA editing of the full-length mRNA transcript. APOB-48 is synthesized in the small intestine and is critical in the assembly of chylomicrons in intestinal enterocytes. APOB-100 is synthesized in the liver and is a major component of very low-density lipoprotein (VLDL), intermediate density lipoprotein, and low-density lipoprotein (LDL). It is important in the formation of VLDL in the liver which has a role in triglyceride export (1) and acts as a ligand for LDL to bind to the LDL receptor (2, 3). Mutations in APOB can cause familial hypobetalipoproteinemia (FHBL) and hypocholesterolemia in human patients (4). Patients with heterozygous mutations of APOB typically have decreased levels of APOB and LDL-cholesterol levels and are predominantly asymptomatic. However, patients with compound heterozygous or homozygous mutations in APOB often display deficiencies in fat-soluble vitamins, intestinal fat malabsorption and neurological problems in addition to having reduced circulating APOB, cholesterol and beta-lipoproteins (2, 4). Furthermore, both heterozygous and homozygous patients have been found to develop metabolic dysfunction-associated steatotic liver disease (MASLD) (1, 5, 6, 7, 8) which can progress to hepatocellular carcinoma (HCC) (9, 10, 11).

While mouse models of Apob deficiency exist (2, 12, 13), they are not ideally suited as a model of human deficiency of APOB as human and mouse lipoprotein profiles are inherently different. In humans, LDL and VLDL are the predominant circulating lipoproteins transporting cholesterol, while in mice, high-density lipoprotein (HDL) has greater prominence (14). As a result, cholesterol levels in mice are much lower than in humans as the majority of cholesterol being transported in HDL. Advantageously, chimeric humanized liver mice have a highly humanized lipoprotein profile including human-specific levels of serum LDL and VLDL relative to serum HDL (15, 16, 17, 18, 19). Thus, these mice provide an ideal platform for studying human liver specific loss of APOB in an in vivo system with a human lipid profile.

Tyrosinemia type I is an inborn error of metabolism caused by a deficiency of fumarylacetoacetate hydrolase (Fah) (20, 21). Fah is an enzyme involved in the tyrosine metabolic pathway and functions in the final step, breaking down fumarylacetoacetate to fumarate and acetoacetate (Fig. 1A). In the absence of Fah, build-up of the toxic metabolite fumarylacetoacetate leads to kidney and liver damage. Patients with tyrosinemia type I are typically treated with the drug 2-(2-nitro-4-trifluoromethylbenzoyl)-1,3-cyclo-hexanedione (NTBC), an inhibitor of 4-hydroxyphenylpyruvic acid dioxygenase (Hpd). Hpd is an upstream enzyme in the tyrosine metabolic pathway, and its inhibition prevents the accumulation of toxic fumarylacetoacetate. Chimeric humanized liver mouse models are created by taking advantage of the Fah-knockout mouse. These humanized mice are generated by transplantation of primary human hepatocytes into *Fah*-deficient (*Fah*^−/−^), immune-deficient (*Rag2*^−/−^, *Il2rγ*^−/−^, *NOD*) mice, termed FRGN (22). The immune deficiency in the FRGN mice prevents the rejection of the transplanted human hepatocytes, and the Fah deficiency allows the transplanted human hepatocytes to have a growth advantage. Fah-deficient mice can be maintained on NTBC to prevent liver failure. Following transplantation of human hepatocytes into the FRGN mice, NTBC is withdrawn. In its absence, *Fah*^−/−^ mouse hepatocytes undergo cell death allowing the human hepatocytes to divide and repopulate the mouse liver (20, 22, 23, 24, 25). These mice can be useful for studying human liver diseases (18, 26), understanding human liver function (27, 28), testing gene editing strategies (26, 29), studying infectious diseases (30, 31, 32, 33, 34) and in vivo testing of drug safety (35). Advantageously, these mice have also been shown to have a highly human lipoprotein metabolic profile, with increased LDL and VLDL fractions and a decreased HDL faction (16) relative to wild-type mice.

**Fig. 1:**
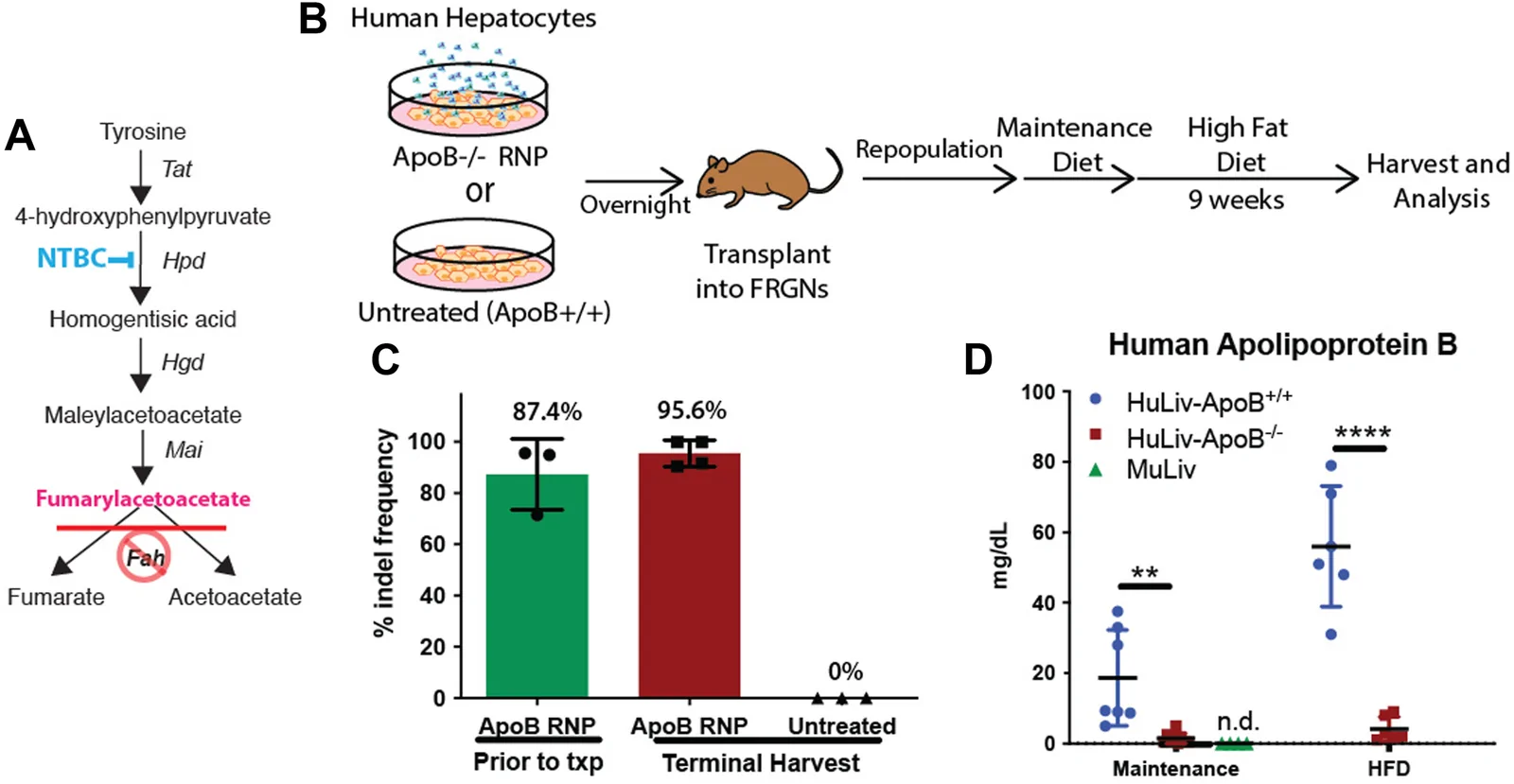
Experimental Procedure and APOB knockout. A: Schematic of the tyrosine metabolic pathway. Knockout of Fah (Fumarylacetoacetate) results in the accumulation of the hepatotoxic byproduct Fumarylacetoacetate. The drug NTBC (2-(2-nitro-4-trifluoromethylbenzoyl)-1,3-cyclo-hexanedione) inhibits an upstream enzyme Hpd (4-hydroxyphenylpyruvic acid dioxygenase), preventing the accumulation Fumarylacetoacetate. *Tat:* Tyrosine aminotransferase; *Hgd*: homogentisate 1,2-dioxygenase; *Mai*: Maleylacetoacetate isomerase. B: Schematic of the experimental procedure. Primary human hepatocytes were treated overnight with SpCas9 protein and a combination of three sgRNAs targeting exon 6 of human APOB. The following morning, the hepatocytes were harvested and transplanted into FRGN mice which were then allowed to repopulate. The mice were then placed on a maintenance diet followed by a high fat diet. C: The insertion/deletion (indel) frequency in APOB prior to transplantation (n = 3) and following terminal harvest (n = 4) of the liver. D: A comparison of circulating human ApoB in the liver repopulated mice while on a maintenance diet and on a high fat diet (n = 6 on each diet). ApoB levels measured in untransplanted FRGN mice (n = 4) was not detectable (n.d.). Data are represented as mean ± SD. ∗*P* < 0.05, ∗∗*P* < 0.01, ∗∗∗*P* < 0.001 and ∗∗∗∗*P* < 0.000.

In this study, we demonstrate that a chimeric humanized liver mouse model with a human hepatocyte-specific APOB deficiency can be generated. This mouse displays phenotypes similar to APOB-deficient patients and demonstrates that human hepatocyte-specific, loss-of-function mutations can be used to create disease models that are superior to standard transgenic mice.

## Materials and methods

### Animals

All animal experiments were performed in accordance with the guidelines for animal care at Oregon Health & Science University. All experiments were approved by the Institutional Animal Care and Use Committee at Oregon Health & Science University. *Fah*^*−/−*^*, Rag2*^*−/−*^*, Il2rγ*^*−/−*^ *NOD* (FRGN) mice (22, 25) were maintained on 8 mg/L of 2-(2-nitro-4-trifluoromethylbenzoyl)-1,3-cyclo-hexanedione (NTBC; Yecuris, Tualatin, OR). Female and male mice aged 5–9 weeks of age were used for hepatocyte transplantation and maintained in group housing. Following transplantation, the mice were cycled on and off 0.1 mg/L and 8 mg/L NTBC in the drinking water as described below. All animals were fed tap either high-energy mouse chow (LabDiet Picolab High Energy Mouse Diet 5LJ5), YF-10: a custom diet containing 0.75% Phenylalanine (Ssniff-Spezialdiaten GmbH, Soest, Germany; see Supplemental Table S1 for detailed dietary composition), or Tekland Custom Diet, TD.120528: a modified western diet containing 1.25% cholesterol (Envigo, Indianapolis, IN). Animals were housed under a standard 12-h on and off light cycle. Mice were euthanized with CO_2_. A cardiac puncture was performed immediately after death to deplete the liver of blood.

### Transplantation of human hepatocytes

Twenty-four to 30 h prior to transplantation, recipient mice were injected via retro-orbital injection with a urokinase expression adenoviral vector (Ad5-uPA) as previously described (22). 350,000 to 500,000 viable human hepatocytes were transplanted into FRGNs by intrasplenic injection as previously described (22). Cryopreserved primary male human hepatocytes (Lot: QIE) were purchased from BioIVT (Woodbury, NY) for transplantation. Following transplantation the mice were subjected to a cycling NTBC withdrawal regimen (3 days on 8 mg/L, 4 days on 0.1 mg/L, 4 days off, 5 days on 8 mg/L, 4 days on 0.1 mg/L, 4 days off, 4 days on 8 mg/L, 3 days on 0.1 mg/L, 4 days off, 4 days on 8 mg/L, 3 days on 0.1 mg/L, 4 days off, 5 days on 8 mg/L, 8 days off, 3 days on 8 mg/L, 7 days off, 3 days on 8 mg/L. Mice were then cycled for 21 days off and 5 days on 8 mg/L until human Albumin levels were above 2.5 mg/ml). Human Albumin levels were measured as previously described to assess the degree of humanization (22) by using human species-specific antibodies against Albumin. Once humanized, the mice were maintained on a custom 0% tyrosine, 0.75% Phenylalanine diet for 6–10 weeks and then switched to a high-fat diet (HFD) for 9 weeks.

### Ribonucleoprotein (RNP) transfection

A combination of three synthetic single guide RNAs (sgRNAs) targeting exon 6 of human *APOB-100*, were designed by and purchased from Synthego. RNPs were assembled by combining the sgRNAs and SpCas9 protein (Integrated DNA Technologies, Coralville, IA) at a 2.5:1 ratio and incubating at room temperature for 10 min. RNPs were placed on ice until ready to use. Transfections were performed in 6-well plates. For each well, 3 μl of RNPs, 12 μl of Cas9plus and 484 μl of Gibco Opti-MEM media (Thermo Fisher Scientific, Waltham, MA) were combined and incubated at room temperature for 5 min 24 μl of Lipofectamine CRISPRMAX Cas9 transfection reagent (Invitrogen, Waltham, MA) and 480 μl of Opti-MEM media was then added and gently mixed by pipetting prior to being incubated at room temperature for 20 min. The transfection mix was then added to one well of a collagen coated 6-well plate (Corning Inc., Corning, NY). 600,000 primary human hepatocytes (BioIVT, Woodbury, NY) resuspended in 2.1 ml of HCM™ Hepatocyte Culture Media (Lonza, Morristown, NJ) were plated on top of the transfection mix and placed into a 37°C, 5% CO_2_ incubator overnight. The next morning, hepatocytes were washed 2× with Gibco DMEM (Thermo Fisher Scientific, Waltham, MA) and then harvested by incubating for 5–10 min with prewarmed 0.25% Gibco Trypsin/EDTA (Thermo Fisher Scientific, Waltham, MA). Hepatocytes were washed 2x in DMEM with 10% FBS, spun at 100 × g for 5 min and resuspended in HCM™ Hepatocyte Culture Media for transplantation.

### Insertion/deletion analysis

Genomic DNA was extracted from homogenized liver tissue using the MasterPure Complete DNA and RNA Purification kit (Lucigen, Middleton, WI) following the manufacturer’s protocol. PCR was performed using MyTaq Red Mix (Meridian Life Science, Memphis, TN) on genomic DNA to amplify a 799 base pair region in human APOB encompassing the PAM site of the 3 sgRNAs (forward primer: 5′-CCTCAGTCCCATGAGCACTTT-3′; reverse primer: 5′-TTTGCTGCCCTACATCCATCC-3′). Primers were designed to be human specific and do not amplify mouse DNA. The PCR products were then cloned into the pCR4-TOPO plasmid using the Invitrogen TA TOPO Cloning kit for Sequencing (Thermo Fisher Scientific, Waltham, MA) following the manufacturer’s protocol then transformed into DH5α competent cells (New England Biolabs Inc., Ipswich, MA) 6 colonies from HuLiv-ApoB^+/+^ animal and 20–25 colonies from HuLiv-ApoB^−/−^ animal were sent for Sanger sequencing using the M13-Forward primer (5′-GTAAAACGACGGCCAG -3′). Alignment of each sequencing result to determine if an insertion or deletion mutation was present was performed using SnapGene Software (Insightful Science LLC, San Diego, CA).

### Immunohistochemistry

Tissues were fixed in 10% Formalin (w/v) at 4°C overnight or 4 h at room temperature and then transferred to 70% ethanol. Tissues were paraffin embedded and the formalin-fixed, paraffin-embedded (FFPE) blocks were sectioned into 5 μm, followed by deparaffination and rehydration. For H&E staining, slides were stained by Carazzi’s hematoxylin (Sigma Aldrich, Saint Louis, MO), followed by eosin-phloxine (Sigma Aldrich, Saint Louis, MO). Sirius red staining was performed using a Picro-Sirius Red Stain Kit (Abcam, #ab150681). For immunostaining, after a heat-induced antigen retrieval, slides were blocked with 3% hydrogen peroxide for 10 min. Subsequently, Slides were permeabilized with 3% Tween-80 for 10 min and blocked with 5% normal goat serum for 1 h. Primary antibodies anti-FAH (Grompe lab) and anti-CD45 (Abcam, #ab10558) were applied for 3 h at room temperature. After washing, the slides were incubated with anti-rabbit HRP for 1 h, and developed using DAB substrate. Slides were counterstained with hematoxylin and hydrated for mounting.

### RNA sequencing

RNA library preparations and sequencing reactions were conducted at GENEWIZ, LLC. Sequencing results are from 5 HuLiv-ApoB^−/−^ and 5 HuLiv-ApoB^-+/+^ mice of mixed sex. RNA samples were quantified using Life Technologies Qubit 2.0 Fluorometer (Thermo Fisher Scientific, Walthma, MA) and RNA integrity was checked using Agilent TapeStation 4200 (Agilent Technologies Inc., Santa Clara, CA).

RNA sequencing libraries were prepared using the NEBNext Ultra RNA Library Prep Kit for Illumina following manufacturer’s instructions (New England Biolabs Inc., Ipswich, MA). Briefly, mRNAs were first enriched with Oligo(dT) beads. Enriched mRNAs were fragmented for 15 min at 94°C. First strand and second strand cDNAs were subsequently synthesized. cDNA fragments were end repaired and adenylated at 3′ends, and universal adapters were ligated to cDNA fragments, followed by index addition and library enrichment by limited-cycle PCR. The sequencing libraries were validated on the Agilent TapeStation (Agilent Technologies, Santa Clara, CA), and quantified by using Qubit 2.0 Fluorometer (Invitrogen, Waltham, MA) as well as by quantitative PCR (KAPA Biosystems, Wilmington, MA).

The sequencing libraries were clustered on flow cells. After clustering, the flow cell was loaded on the Illumina HiSeq instrument (4000 or equivalent) according to manufacturer’s instructions. The samples were sequenced using a 2x150 bp Paired End (PE) configuration. Image analysis and base calling were conducted by the HiSeq Control Software (HCS) version HD 3.5.0.7. Raw sequence data (.bcl files) generated from Illumina HiSeq was converted into fastq files and demultiplexed using Illumina's bcl2fastq 2.17 software. One mismatch was allowed for index sequence identification.

The transcriptomic and differential gene expression data generated from this study is available at Gene Expression Omnibus using the accession number GSE31556.

### Bioinformatic analysis

FASTQ files of RNA-Seq data were processed using nf-core/rnaseq v3.14.0 (36) of the nf-core collection of workflows (37), utilizing reproducible software environments from the Biocontainers project (38). The pipeline was executed with Nextflow v23.10.1 (39). A summary of the data processing workflow is as follows: sequencing reads were mapped against both human GRCh38 and mouse GRCm38 using the disambiguation tool bbsplit (40). Human reads were subjected to quality control with FastQC (41) and then aligned with STAR (42). Aligned reads were quantified with RSEM (43). Quantified reads were subjected to differential gene expression analysis with limma (44) and gene set enrichment analysis with fgsea (45) using the R 4.4 (46) and Bioconductor 3.19 (47) analysis environments.

### Blood and intrahepatic lipid analysis

Blood triglycerides, cholesterol, HDL, and LDL analytes were assayed in a Siemens ADVIA Chemistry XPTB Clinical System. APOB was measured using the ADVIA Chemistry XPTB Clinical system using Siemen’s ADVIA Chemistry APO-B buffer and Siemen’s ADVIA Chemistry APOB antibody reagent. Both reagents were designed for testing APOB levels in human serum and plasma. Triglyceride and cholesterol measurements in the liver tissue were measured by Infinity Triglyceride, Infinity Cholesterol reagent (Thermo Fisher Scientific, Walthma, MA) and NEFA reagent (Wako Diagnostics, Richmond, VA), respectively. For these measurements, at terminal harvest three small pieces of liver tissue were collected from three lobes of the liver for a total weight of 60–80 mg. The liver pieces were immediately frozen on dry ice. Prior to analysis the livers were processed by bead homogenization in lysing matrix D tubes using the FastPrep-24 5G homogenizer (MP Biomedical, Irvine, CA) in water. The homogenate was then extracted for lipids using the Folch method. Vitamin E was measured using mass spectrometry by IDEXX Laboratories (Westbrook, ME).

### Western blots

Flash frozen livers were homogenized in lysis buffer containing protease inhibitors. 20 μg of lysate was loaded onto appropriate percentage SDS-PAGE gels and proteins were separated by gel electrophoresis. After separation proteins were transferred to polyvinylidene fluoride membranes. The membrane was incubated with primary antibody overnight. Primary antibodies used included LC3B (Cell Signaling Technology catalog #83506, clone E5Q2K, used at 1:1,000), ApoB (Proteintech catalog #20578-1-AP, used at 1:1,000) and GAPDH (Origene catalog #TA802519, clone 2D9, used at 1:5,000). The membrane was then incubated with the corresponding HRP-conjugated secondary antibody. The proteins were detected for visualization using SuperSignal West Pico PLUS (Thermo Fisher Scientific, Waltham, MA). Membranes were imaged using the BioRad ChemiDoc Touch Imaging System.

## Results

To create a model of human APOB deficiency, mice with APOB-deficient chimeric livers were generated. Primary human hepatocytes were transfected ex vivo with CRISPR/spCas9 RNPs. A combination of three sgRNAs targeting exon 6 of *APOB-100* was used. After 18–20 h of plating following RNP transfection, immune-deficient Fah knockout (FRGN) mice (25) were transplanted with *APOB* RNP-transfected hepatocytes or untransfected, unmanipulated control hepatocytes from the same donor (Fig. 1B). These mice will be referred to as HuLiv-ApoB^−/−^ and HuLiv-ApoB^+/+^, respectively, from here forward. Upon transplantation of (*Fah*^+/+^) human hepatocytes, withdrawal from NTBC results in repopulation of the FRGN mouse liver by human hepatocytes (22). Circulating levels of human Albumin, which is produced by the human hepatocytes (22, 25), were measured by utilizing a human-specific Albumin antibody to estimate the degree of liver repopulation by human hepatocytes. Both groups of mice were confirmed by measurement of circulating human Albumin concentrations to have sufficient levels of human hepatocyte repopulation prior to further analysis of their phenotypes (Supplemental Fig. S1A, B). Immunohistochemistry of liver sections following terminal harvest for Fah further confirmed humanization of the liver. As the FRGN mice are Fah knockout, only human hepatocytes stain positive for FAH (Supplemental Fig. S1C–E).

To determine the effectiveness of the *APOB* targeting RNPs in knocking out the target gene, insertion/deletion (indel) mutation analysis was done. To measure the indel frequency of transplanted cells, a subset of the *APOB* RNP-treated human hepatocytes were plated in tissue culture for 5 days after RNP treatment. The *APOB* indel frequency in the plated hepatocytes was 87.4% ± 13.8% (n = 3). At terminal harvest of repopulated mice, the indel frequency in HuLiv-ApoB^−/−^ mice was 95.5% ± 5.2% (n = 4) and 0% (n = 3) in HuLiv-ApoB^+/+^ mice (Fig. 1C). These frequencies indicate highly efficient cleavage by the APOB-targeting spCas9 RNPs and indicate that the absence of any growth advantage or disadvantage of the APOB deficient human hepatocytes during the expansion and repopulation of the liver. To confirm the disruption and loss of function of APOB, circulating human APOB levels were measured using a species-specific ELISA. In HuLiv-APOB^+/+^ mice, average circulating APOB was 18.63 mg/dl on the maintenance diet and 56.00 mg/dl on the high fat, high-cholesterol diet with 3.5% w/v dextrose water (HFD). In contrast, human APOB levels were negligible in the HuLiv-ApoB^−/−^ mice (Fig. 1D), confirming the disruption of the APOB protein. Untransplanted control FRGN mice (n = 4) had undetectable levels of APOB, confirming that the APOB assay was human specific (Fig. 1D).

Immunoblotting for APOB demonstrated a significant decrease (*P* = 0.0162) of ApoB-100 in the HuLiv-ApoB^−/−^ livers (n = 3) as compared to the HuLiv-ApoB^+/+^ livers (n = 3) (Supplemental Fig. S1F, G), confirming a loss of most ApoB-100 expression. Human hepatocytes only express APOB-100, while mouse hepatocytes express both isoforms of ApoB-100 and ApoB-48 (48, 49, 50). Similar levels of ApoB-48 were seen in the HuLiv-ApoB^−/−^ and the HuLiv-ApoB^+/+^ mice, suggesting that this isoform was produced mostly by mouse hepatocytes. Interestingly, the ApoB-48 levels were clearly elevated in both groups of humanized mice relative to untransplanted FRGN mice (Supplemental Fig. S1FH). This suggests that NTBC withdrawal resulted in the upregulation of ApoB-48 expression in the residual mouse hepatocytes present in the HuLiv-ApoB^−/−^ and the HuLiv-ApoB^+/+^ mice livers.

To determine the effects of loss of APOB on circulating lipid levels, blood lipid analysis was performed in the highly liver humanized mice. Analysis of HuLiv-ApoB^+/+^ mice and HuLiv-ApoB^−/−^ mice was first performed while the mice were on a regular diet and subsequently after being placed on a HFD for 9 weeks. While the APOB levels measured were human-specific, other blood lipid measurements were not species-specific and represent the combined amount of mouse and human metabolites. On a maintenance diet there was a significant decrease in circulating LDL cholesterol (*P* < 0.0001) (Fig. 2A) and triglycerides (*P* = 0.003) (Fig. 2B) but no significant differences in total cholesterol (*P* = 0.124) (Fig. 2C) and HDL cholesterol (*P* = 0.074) (Fig. 2D) in the HuLiv-ApoB^−/−^ mice compared to the HuLiv-ApoB^+/+^ mice.

**Fig. 2:**
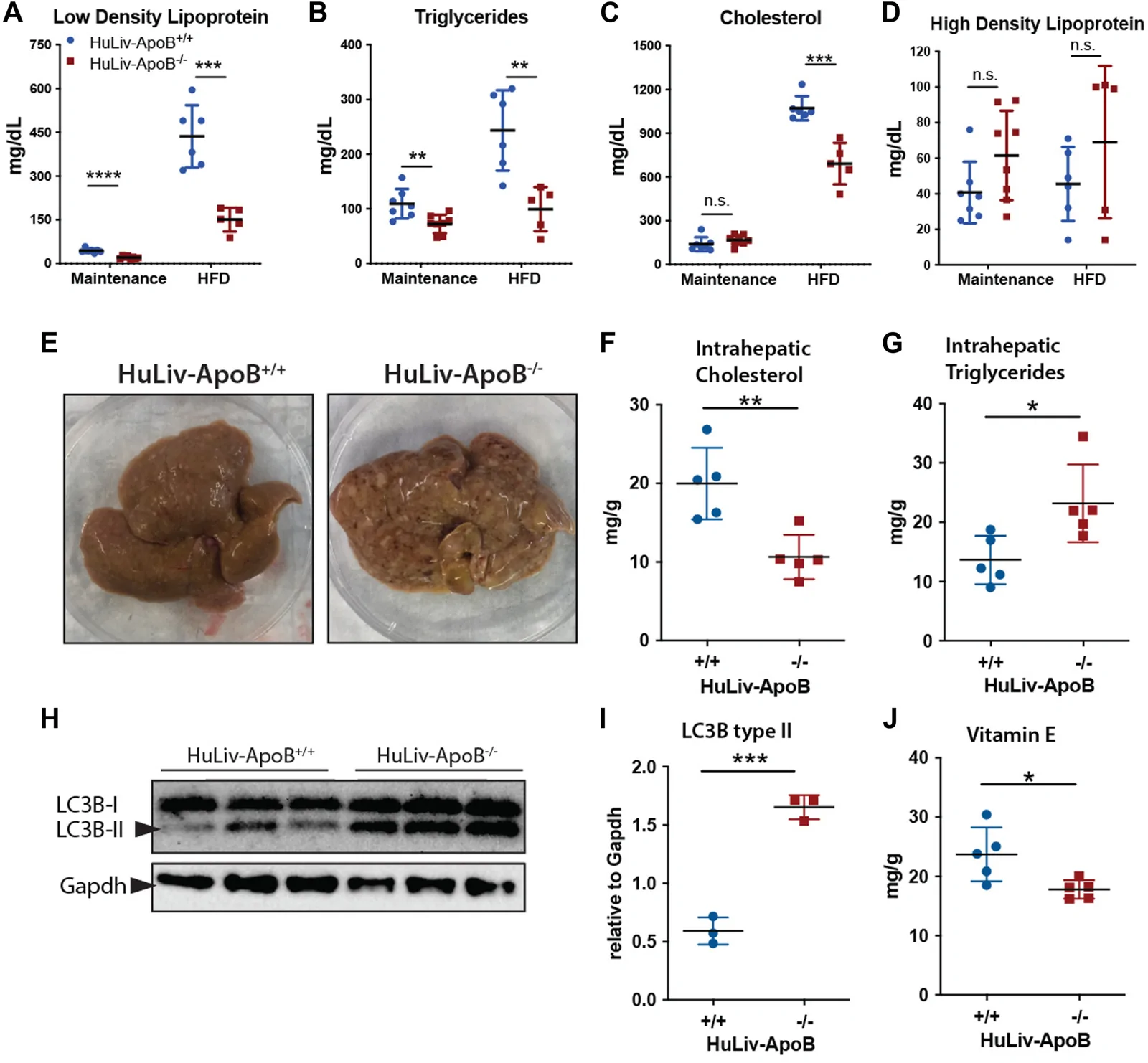
Circulating and Intrahepatic lipid measurements. Circulating (A) low-density lipoprotein, (B) triglycerides, (C) Cholesterol, and (D) high-density lipoprotein in the humanized mice either on a maintenance (n = 7 per group) or high fat (HFD) (n = 5 per group). E: Representative images of whole liver from terminally harvested mice maintained on HFD. (F) Intrahepatic cholesterol content at terminal harvest from HuLiv-ApoB^−/−^ (n = 5) and HuLiv-ApoB^+/+^ livers (n = 5). G: Intrahepatic triglyceride content at terminal harvest from HuLiv-ApoB^−/−^ (n = 5) and HuLiv-ApoB^+/+^ livers (n = 5). H: Immunoblots for LC3B and Gapdh from liver homogenates. LC3B-II and GAPDH are indicated with arrow heads. I: Relative concentration of LC3B-II present in the livers (n = 3 per group). J: Circulating levels of vitamin E at terminal harvest measured from serum (n = 5 from each group). Data are represented as mean ± Standard Deviation. ∗*P* < 0.05, ∗∗*P* < 0.01, ∗∗∗*P* < 0.001 and ∗∗∗∗*P* < 0.0001.

Patients with a loss of APOB typically have depressed levels of total cholesterol (1). While lowered amounts of LDL cholesterol were observed in the HuLiv-ApoB^−/−^ mice, total cholesterol remained unaffected on a regular diet. To determine whether lowered levels of cholesterol could be modeled in the HuLiv-ApoB^−/−^ mice, the mice were placed on a HFD. After 9 weeks on the HFD, both the HuLiv-ApoB^+/+^ and HuLiv-ApoB^−/−^ mice had increased LDL cholesterol (Fig. 2A), triglycerides (Fig. 2B) and total cholesterol (Fig. 2C) compared to their levels while on a maintenance diet. Although an overall increase of blood lipids were observed, HuLiv-ApoB^−/−^ mice displayed significantly lower amounts of circulating LDL cholesterol (*P* = 0.0003), triglycerides (*P* = 0.004) and total cholesterol (*P* = 0.0004) as compared to the HuLiv-ApoB^+/+^ mice, thus demonstrating that the HuLiv-ApoB^−/−^ mice were more resistant to increases in LDL cholesterol and total cholesterol typically induced by a HFD.

ApoB plays a role in the rate-determining step for lipid export from hepatocytes (51, 52, 53) and loss of function mutations in ApoB results in decreased plasma cholesterol levels. We hypothesized that the decreased amount of circulating cholesterol in the HuLiv-ApoB^−/−^ mice was due to accumulation of the lipids within the hepatocytes. Macroscopically, at harvest, large white areas indicating lipid accumulation were readily observed in all mice repopulated with knockout hepatocytes (Fig. 2E). Intrahepatic cholesterol and triglyceride content were analyzed. Significantly increased levels of cholesterol (Fig. 2F) were present in the hepatocytes of HuLiv-ApoB^−/−^ mice compared to the untreated HuLiv-ApoB^+/+^ mice, suggesting that there was a failure of cholesterol export from hepatocytes as expected. Additionally, a decreased amount of intrahepatic triglycerides (Fig. 2G) in the HuLiv-ApoB^−/−^ mice compared to the HuLiv-ApoB^+/+^ mice was found. This finding was unanticipated as an accumulation of intrahepatic triglycerides was expected in the absence of ApoB. When Conlon *et al.* (54) utilized antisense oligonucleotides to knock out ApoB in mice, they also found that there was lack of hepatic lipid accumulation due to a compensatory increase of autophagy of the endoplasmic reticulum. To investigate whether this also explained the lack of triglyceride accumulation in our HuLiv-ApoB^−/−^ mice, a subset of liver samples were probed by Western blot for levels of microtubule-associated protein 1 A/1B light chain isoform 3B type II (LC3B-II). LC3B-II is a marker of autophagy and is the lipidated form of LC3B-I required in the formation of the autophagosomal membrane (55). We observed a significant (*P* = 0.0003) increase in the amount of LC3B-II present in the HuLiv-ApoB^−/−^ livers (n = 3) relative to the HuLiv-ApoB^+/+^ livers (n = 3) (Fig. 2H, I), consistent with the induction of autophagy.

Another common finding in human APOB-deficient patients is a deficiency of fat-soluble vitamins A and E. This deficiency is variable, dependent on the patient’s particular APOB mutation, and, in extreme cases, can result in progressive ophthalmopathy and ataxic neuropathy. These symptoms can be slowed with long-term high-dose vitamin A and E supplementation starting at a young age (56, 57). Vitamin E was measured in our humanized liver mice to determine whether they display the same deficiencies. In HuLiv-ApoB^−/−^ mice (n = 5) there was on average 17.80 ng/ml circulating vitamin E in the blood, relative to 23.73 ng/ml in HuLiv-ApoB^+/+^ mice (n = 5). Levels of vitamin E were significantly lowered in HuLiv-ApoB^−/−^ mice compared to the untreated HuLiv-ApoB^+/+^ mice (*P* = 0.0246) (Fig. 2J) reflecting the same phenotype as observed in human patients.

A homozygous loss of APOB in patients can lead to the development of MASLD (10). To determine whether our mice displayed features of MASLD, histological analysis was performed. In both the HuLiv-ApoB^+/+^ and HuLiv-ApoB^−/−^ mice, H&E staining revealed minor steatosis with some ballooning of hepatocytes and lipid accumulation (Supplemental Fig. S2A). Fibrosis of the liver begins to occur at the onset of MASLD (58). To determine whether the HuLiv-ApoB^−/−^ mice had begun to have these lesions, staining against CD45 and Sirius red was performed. No differences in CD45 and Sirius red staining were observed in both the HuLiv-ApoB^−/−^ and HuLiv-ApoB^+/+^ mice (Supplemental Fig. S2B, C).

Following terminal harvest RNA sequencing was performed on a subset of the samples to evaluate the effects of a loss of human APOB on other gene regulation pathways. Despite the strong phenotypical differences observed in circulating lipid levels and hepatic cholesterol accumulation, the human gene expression profiles of the HuLiv-ApoB^−/−^ (n = 5) hepatocytes were mostly similar compared to the HuLiv-ApoB^+/+^ (n = 4). Differential human gene expression analysis was performed comparing HuLiv-ApoB^−/−^ to HuLiv-ApoB^+/+^ livers and top 20 differentially genes are shown in a heatmap (Fig. 3). Analysis revealed that out of the 11,425 gene transcripts, only 16 genes that had significant differential expression between the two groups (-log10 adjusted *P*-value >1.25). Two of those genes, Microtubule-associated protein 2 (MAP2) and Nuclear receptor subfamily 1 group D member 1 (NR1D1) had increased expression in the livers of HuLiv-ApoB^−/−^ mice compared to the livers HuLiv-ApoB^+/+^ mice while the remaining 12 genes had decreased expression. Further analysis revealed that there were no signs of significant upregulation or down regulation of any lipid metabolism relevant gene pathways. Analysis of human *APOB* gene expression demonstrated that there was lowered expression in the HuLiv-ApoB^−/−^ livers as compared to the HuLiv-ApoB^+/+^ livers. Although this difference was not significant, it correlates with a gene knockout as mRNA may still be transcribed from the locus (Fig. 3B). Hence, while differences are observed in circulating cholesterol and lipids in HuLiv-ApoB^−/−^ livers, no large-scale changes in human gene expression occurred.

**Fig. 3:**
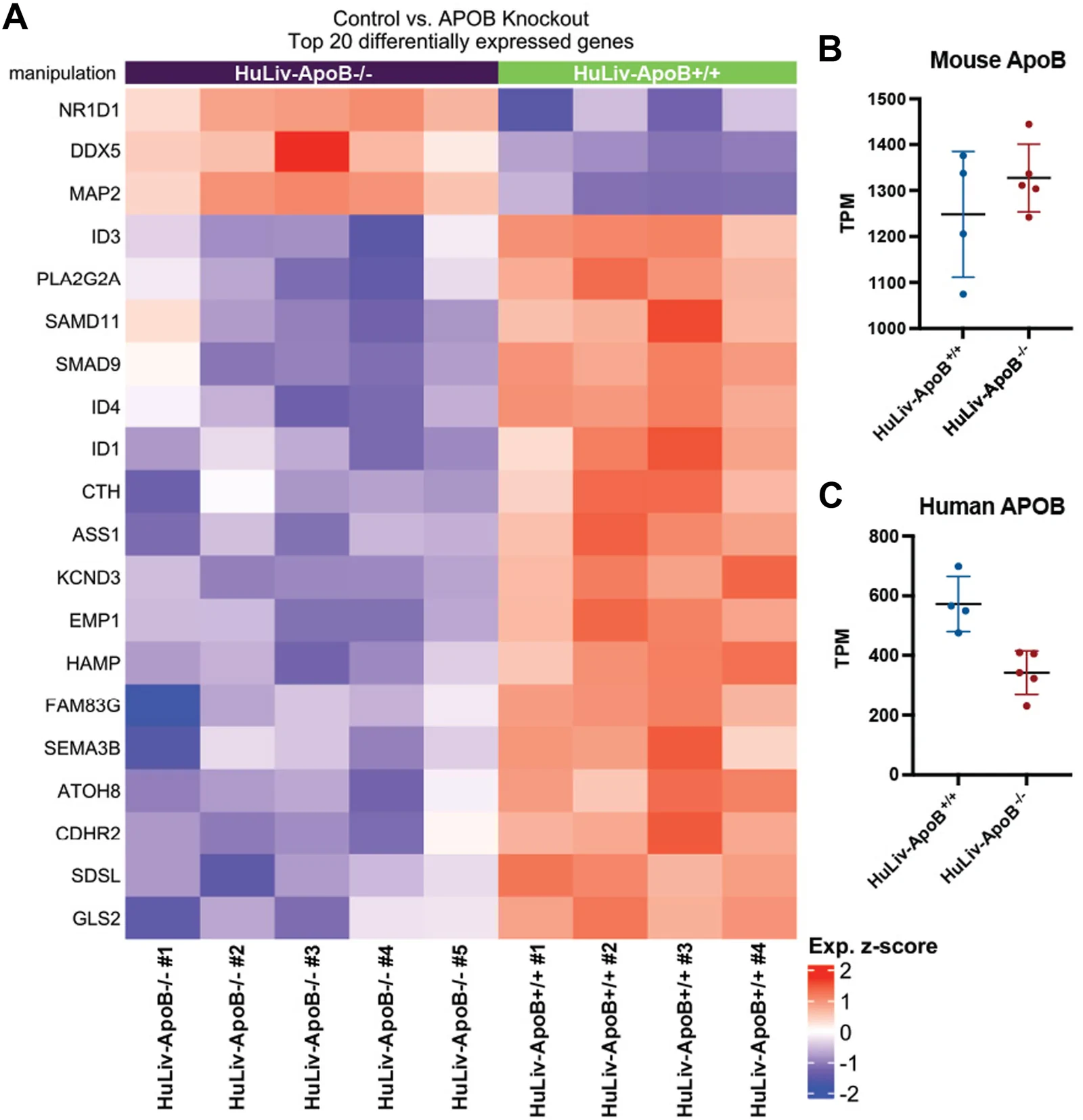
Gene expression analysis by RNA sequencing. A: A heatmap showing differential human gene expression from HuLiv-ApoB^−/−^ (n = 5) and HuLiv-ApoB^+/+^ livers (n = 4). B: A comparison of mouse APOB expression levels and (C) human APOB expression levels from the RNA sequencing results of the livers of HuLiv-ApoB^−/−^ and HuLiv-ApoB^+/+^. TPM: Transcripts per million.

To determine whether the residual mouse hepatocytes were regulating their gene expression patterns in response to the presence of human hepatocytes with a loss of APOB, analysis of the mouse gene expression data was performed. Of a total of 14,260 gene transcripts, only 40 genes had significant differential expression between the two groups (-log10 adjusted *P*-value >1.25). There were no significant differences in *APOB* gene expression levels between HuLiv-ApoB^−/−^ livers as compared to the HuLiv-ApoB^+/+^ livers (Fig. 3C), consistent with our Western blot results. This indicates that no compensation occurred in response to the lack of human APOB.

## Discussion

The experiments reported here are the first to demonstrate ex vivo gene editing of primary human hepatocytes, followed by expansion in an immune compromised Fah-deficient mouse model. We demonstrated that a humanized liver mouse model of familial hypobetalipoproteinemia can be created. The use of CRISPR/spCas9 RNPs to disrupt human *APOB* resulted in a >95% indel mutation frequency, corresponding to an almost complete loss of circulating human APOB-100 protein. Ex-vivo knockout of a human gene by using a combination of three CRISPR/spCas9 RNPs in primary hepatocytes was highly efficient as shown by indel analysis prior to transplantation and also after liver repopulation. Immunoblotting for ApoB further confirmed that at the protein levels of ApoB-100 were significantly decreased in the HuLiv-ApoB^−/−^ mice. Levels of murine ApoB-48 were unaffected by the APOB knockout of human hepatocytes.

The blood lipid phenotypes observed in the HuLiv-ApoB^−/−^ mouse paralleled those of human familial hypobetalipoproteinemia patients. In HuLiv-ApoB^−/−^ mice, LDL cholesterol and triglycerides were decreased, while HDL cholesterol remained unaffected. ApoB is an important component of VLDL and LDL and is crucial for the export of triglycerides and cholesterol from the liver (51, 52, 53). With a deficiency in ApoB, the expected decrease in circulating triglycerides due to the impairment in lipid export was observed. While the assay for APOB was human-specific, cholesterol measurements are not. LDLs are formed when triglycerides are removed from VLDLs and are transported into the fat or muscle (59). Due to the impaired export of VLDLs, there was also an observed decrease in circulating LDLs, and analysis of intrahepatic lipid content confirmed a significant increase in total cholesterol.

As there was a decrease in circulating LDLs, triglycerides and cholesterol in HuLiv-ApoB^−/−^ mice, an increase in intrahepatic lipid accumulation was expected. While the expected increase in intrahepatic cholesterol was observed in HuLiv-ApoB^−/−^ mice, a decrease in intrahepatic triglycerides was found. Previous studies by others have shown that a loss of ApoB in vivo by use of antisense oligonucleotides lead to decreased circulating cholesterol and triglycerides without increases in levels of intrahepatic triglycerides or the development of hepatic steatosis (54, 60). A study by Conlon *et al.* (54) in a mouse model treated with ApoB antisense oligonucleotides for 6 weeks showed that the increase of lipid droplet accumulation in the endoplasmic reticulum (ER) activates ER autophagy and increased fatty acid oxidation in response to the inability of the hepatocyte to secrete VLDLs. This resulted in lowered intrahepatic triglyceride content and absence of steatosis. Here, Western blot analysis also showed HuLiv-ApoB^−/−^ mice also had increased levels of LC3B-II, a marker of autophagy, as compared to the HuLiv-ApoB^+/+^ mice. Therefore, a likely explanation for the lack of intrahepatic triglyceride accumulation in the HuLiv-ApoB^−/−^ mice is the increase in autophagy. Additionally, when gene expression analysis by qRT-PCR was performed in the Conlon *et al.* study to analyze genes involved in hepatic lipid homeostasis, it was found that genes involved in the pathways related to lipid synthesis, hepatic uptake of fatty acids and fatty acid oxidations were not consistently differentially expressed. Similar results were observed from the RNA-seq analysis of the low number of differentially expressed genes in the HuLiv-ApoB^−/−^ mice as compared to the HuLiv-ApoB^+/+^ mice. An in vitro study by Valmiki *et al.* (61) knocked out APOB in Huh7 cell lines. No accumulation of triglycerides was histologically observed in the knockout cells and quantitative measurements showed no significant difference in triglyceride levels between the knockout and control cells.

Mipomersen (Kynamro) is an antisense oligonucleotide drug that functions by inhibiting APOB-100. It was previously available to patients for treatment of familial hypercholesterolemia (62) but has been discontinued due to risk of hepatotoxicity (63). During clinical trials, patients on the drug were found to have significantly lowered circulating APOB, LDLs, and triglycerides compared to baseline (64, 65) similar to the phenotypes observed in the HuLiv-ApoB^−/−^ mice. While some of the clinical studies report that the majority of patients had no changes in intrahepatic lipid content compared to the placebo group (64), others have reported increases in liver transaminases and that hepatic steatosis without inflammation was observed when Mipomersen was used long term (66). These clinical trials are somewhat different to what we have observed in the HuLiv-ApoB^−/−^ mice.

In this study, HFD was utilized to exacerbate the dyslipidemia phenotype. Mice maintained on a HFD mice typically display dyslipidemia, where significant increases in serum cholesterol and serum HDL cholesterol, as well as slight increases in serum triglycerides, are observed (67, 68, 69). When on a HFD long term, these mice typically develop steatosis which can progress to hepatocellular carcinoma. As expected, in our experiments the HuLiv-ApoB^+/+^ and HuLiv-ApoB^−/−^ mice both displayed overall increases in circulating cholesterol, LDL and triglycerides on a HFD diet as compared to regular chow. The HuLiv-ApoB^−/−^ mice had smaller changes in their increase of LDL cholesterol and total cholesterol as compared to the HuLiv-ApoB^+/+^ mice.

Despite the clear-cut biochemical differences between animals repopulated with APOB knockout and control human hepatocytes, only minimal differences in hepatocyte gene expression in both human and mouse cells were found. While there was a strong circulating lipid phenotype observed in the HuLiv-ApoB^−/−^ mice, gene expression analysis and histology results were consistent in demonstrating that a loss of APOB in the human hepatocytes does not cause increased fibrosis or steatosis in the liver during the experimental time frame used. Although these mice were maintained on a high-fat diet for 9 weeks, it is possible that a longer time period may be required for the development of MASLD. In addition, MASLD and fibrosis have a strong inflammatory component (70, 71) which may be difficult to recapitulate in immune deficient mice. It is also possible that a higher degree of human hepatocyte repopulation than achieved here is necessary to fully recapitulate the MASLD phenotype.

In these experiments, human hepatocytes were derived from a single donor, and it is possible that variability in results may be observed when utilizing different donors. Others have demonstrated that genetic mutations in PNPL3 (18) and TM6SF2 can lead to MASLD (72). Hence, for future experiments generating an APOB knockout model, donor hepatocytes should be prescreened for potential genetic causes of steatosis. An additional limitation of this study is that levels of liver repopulation with human hepatocytes were somewhat lower in HuLiv-ApoB^−/−^ mice relative to mice transplanted with unmanipulated control cells (Supplemental Fig. S1). It is unknown whether the loss of APOB itself or the ex vivo manipulation of the cells with CRISPR/spCas9 RNPs is responsible for this effect. Despite this limitation, both groups achieved high levels of liver repopulation and the HuLiv-ApoB^−/−^ mice showed known phenotypes associated with a loss of APOB.

Generating this mouse model of HuLiv-ApoB^−/−^ human hepatocytes can be important for testing therapies for the treatment of patients with genetically acquired hypobetalipoproteinemia, as there is a high unmet therapeutic need. Gene therapy by gene addition of full-length *APOB* cDNA using vectors such as adeno-associated viruses (73, 74) or lipid nanoparticles can restore the gene expression of APOB in patients. Alternatively, cell therapy alongside selective expansion of transplanted wild-type hepatocytes (75) could restore functional APOB in the liver. In our humanized liver mouse model of APOB deficiency presented here, we have demonstrated that transplantation of human hepatocytes with a lipid metabolism disorder is able to sustainably maintain a humanized lipid profile in the mouse in vivo. This opens the door for the establishment of additional models of other genetically acquired lipid metabolism disorders and to study potential therapies for them. These could include gene therapies, cell therapies and small molecule drugs.

While we here demonstrated a loss-of-function phenotype by creating DNA double-strand breaks in the above experiments to create a disease model, it is foreseeable that a similar strategy with base or prime editors could be also utilized to correct or introduce disease causing mutations in donor hepatocytes. Gene editing and expansion of primary human hepatocytes has previously been demonstrated using either ex vivo lentiviral transduction followed by transplantation (22) or ex vivo rAAV gene editing and tissue culture expansion of hepatocytes (24). Lipid transduction of RNPs is a nonviral delivery method compared to these alternative methods, which both utilize viral transduction. The use of a viral vector can lead to an increased chance of off-target integration and toxicity to the hepatocytes (25).

To summarize, the HuLiv-ApoB^−/−^ mouse model generated in this study provides a platform to increase the understanding of a deficiency in APOB and test possible therapies. By utilizing a chimeric humanized liver mouse as a model in place of a knockout mouse, the gene knockout is localized to hepatocytes circumventing any embryonic lethality that can result from a whole organism knockout of APOB (76). Additionally, ex vivo gene editing is advantageous when studying diseased alleles in a humanized liver mouse as it does not require donor hepatocytes from a diseased patient. Any human gene of interest can be targeted with RNPs with our approach. Our work serves as a proof-of-principle that knockout humanized liver mice can be created to study genetic causes underlying dyslipidemias and metabolic diseases.

## Data availability

Data generated from these experiments are reported in this manuscript and the corresponding supplemental data file. The transcriptomic and differential gene expression data generated from this study is available at Gene Expression Omnibus under the accession number GSE315563 or through this direct link: https://urldefense.com/v3/__https://www.ncbi.nlm.nih.gov/geo/query/acc.cgi?acc=GSE315563__;!!Mi0JBg!MY6-lWnJeYqjzIiFLPMZQDSeQrytf1V04lNmd51w4_ZPjpzgEuS9I95CjTlAedP6dJ65PrFApaoV_LnnWtc$.

## Supplemental data

This article contains supplemental data.

## Conflict of interests

The authors declare the following financial interests/personal relationships which may be considered as potential competing interests:

M. Grompe has a financial interest in Yecuris Corp. (Tigard, Oregon), a company that has commercialized the humanized mouse liver model used in this work. M.Grompe is also a consultant for Cytotheryx, Inc. X. Cheng and H. Chen are employees of Regeneron Pharmaceuticals.
