# Supplementary material for: A humanized mouse model of APOB deficiency recapitulates hypobetalipoproteinemia
Source: J Lipid Res. 2026 Sep 2;67(10):101142. doi: 10.1016/j.jlr.2026.101142 (PMC13658873; doi:10.1016/j.jlr.2026.101142)
Supplement: Supplementary file 1 [file mmc1.docx]

**Supplemental Data**

**A humanized mouse model of APOB deficiency recapitulates Hypobetalipoproteinemia**

Amita Tiyaboonchai, Anne Vonada, Jeffrey Posey, Kevin L. Keys, Carl Pelz, Helen Chen, Xiping Cheng and Markus Grompe

**Supplemental Table S1**

**Composition of the custom 0.75% Phenylalanine Diet**

**Supplemental Table S1: Composition of the maintenance diet.**

**Supplemental Figure S1**

**Supplemental Figure S1: Liver humanized mice.** Circulating levels of human Albumin in HuLiv-ApoB^-/-^ and HuLiv-ApoB^+/+^ mice (A) on a maintenance diet and (B) after 9 weeks on a high fat, high cholesterol diet. Human Albumin >3,000 ug/mL indicate a highly (>60%) humanized liver. (C) Representative images from immunohistology of liver sections stained against Fah. Only human hepatocytes are Fah positive. (D) Images of liver sections that were stained for Fah at higher magnification. (E) Percentage of liver sections that stain positive for Fah were quantified confirming the repopulation of the mice with human hepatocytes. (F) Immunoblotting of liver homogenates from HuLiv-ApoB^-/-^ mice, HuLiv-ApoB^+/+^ mice and untransplanted FRGN mice for APOB. (G) Quantification of ApoB-100 relative to total loaded protein. (H) Quantification of ApoB-48 relative to total loaded protein.

**Supplemental Figure S2**

**Supplemental Figure S2: Histology from HuLiv-ApoB^-/-^ mice and HuLiv-ApoB^+/+^ mice livers.** Representative images of liver sections from HuLiv-ApoB^-/-^ mice and HuLiv-ApoB^+/+^ mice stained for (A) Sirius Red, (B) CD45 and (C) H&E. Scale bar is 100 μm, all images were taken at the same magnification.
